# Clinical course of patients with adrenal incidentalomas and cortisol autonomy: a German retrospective single center cohort study

**DOI:** 10.3389/fendo.2023.1123132

**Published:** 2023-05-08

**Authors:** Hanna Remde, Stefanie Kranz, Sarah Maria Morell, Barbara Altieri, Matthias Kroiss, Mario Detomas, Martin Fassnacht, Timo Deutschbein

**Affiliations:** ^1^ Department of Internal Medicine I, Division of Endocrinology and Diabetes, University Hospital, University of Würzburg, Würzburg, Germany; ^2^ Department of Dermatology and Allergology, University Hospital Augsburg, Augsburg, Germany; ^3^ Department of Nephrology, Agaplesion Markus Krankenhaus, Frankfurt, Germany; ^4^ Department of Internal Medicine IV, University Hospital, Ludwig Maximilians University Munich, Munich, Germany; ^5^ Medicover Oldenburg MVZ, Oldenburg, Germany

**Keywords:** adrenal imaging, adrenal tumours, autonomous cortisol secretion, cardiovascular events, cardiovascular risk factors, dexamethasone suppression test, morbidity, mortality

## Abstract

**Background:**

Adrenal incidentalomas with cortisol autonomy are associated with increased cardiovascular morbidity and mortality. Specific data on the clinical and biochemical course of affected patients are lacking.

**Methods:**

Retrospective study from a tertiary referral centre in Germany. After exclusion of overt hormone excess, malignancy and glucocorticoid medication, patients with adrenal incidentalomas were stratified according to serum cortisol after 1 mg dexamethasone: autonomous cortisol secretion (ACS), >5.0; possible ACS (PACS), 1.9-5.0; non-functioning adenomas (NFA), ≤1.8 µg/dl.

**Results:**

A total of 260 patients were enrolled (147 women (56.5%), median follow-up 8.8 (2.0-20.8) years). At initial diagnosis, median age was 59.5 (20-82) years, and median tumour size was 27 (10-116) mm. Bilateral tumours were more prevalent in ACS (30.0%) and PACS (21.9%) than in NFA (8.1%). Over time, 40/124 (32.3%) patients had a shift of their hormonal secretion pattern (NFA to PACS/ACS, n=15/53; PACS to ACS, n=6/47; ACS to PACS, n=11/24; PACS to NFA, n=8/47). However, none of the patients developed overt Cushing’s syndrome. Sixty-one patients underwent adrenalectomy (NFA, 17.9%; PACS, 24.0%; ACS, 39.0%). When non-operated patients with NFA were compared to PACS and ACS at last follow-up, arterial hypertension (65.3% vs. 81.9% and 92.0%; p<0.05), diabetes (23.8% vs. 35.6% and 40.0%; p<0.01), and thromboembolic events (PACS: HR 3.43, 95%-CI 0.89-13.29; ACS: HR 5.96, 95%-CI 1.33-26.63; p<0.05) were significantly less frequent, along with a trend towards a higher rate of cardiovascular events in case of cortisol autonomy (PACS: HR 2.23, 95%-CI 0.94-5.32; ACS: HR 2.60, 95%-CI 0.87-7.79; p=0.1). Twenty-five (12.6%) of the non-operated patients died, with higher overall mortality in PACS (HR 2.6, 95%-CI 1.0-4.7; p=0.083) and ACS (HR 4.7, 95%-CI 1.6-13.3; p<0.005) compared to NFA. In operated patients, prevalence of arterial hypertension decreased significantly (77.0% at diagnosis to 61.7% at last follow-up; p<0.05). The prevalence of cardiovascular events and mortality did not differ significantly between operated and non-operated patients, whereas thromboembolic events were significantly less frequent in the surgical treatment group.

**Conclusion:**

Our study confirms relevant cardiovascular morbidity in patients with adrenal incidentalomas (especially those with cortisol autonomy). These patients should therefore be monitored carefully, including adequate treatment of typical cardiovascular risk factors. Adrenalectomy was associated with a significantly decreased prevalence of hypertension. However, more than 30% of patients required reclassification according to repeated dexamethasone suppression tests. Thus, cortisol autonomy should ideally be confirmed before making any relevant treatment decision (e.g. adrenalectomy).

## 1 Introduction

Mild hypercortisolism without clinical features of Cushing’s syndrome is a common finding among patients with adrenal incidentalomas (1). This condition, historically been referred to as “subclinical Cushing’s syndrome”, is nowadays termed “cortisol autonomy” (as it represents an own entity and not an early form of clinically overt Cushing’s syndrome) (2).

According to the serum cortisol levels after an overnight 1-mg dexamethasone suppression test (DST), patients are stratified into three study subgroups: autonomous cortisol secretion (ACS), >5.0 µg/dl; possible ACS (PACS), 1.9-5.0 µg/dl; non-functioning adenoma (NFA), ≤1.8 µg/dl (1). The clinical relevance of PACS and ACS remained an object of controversies for a long time (3–5). In recent years, however, an accumulating number of studies have indicated a higher prevalence not only of cardiovascular risk factors but also of cardiovascular events in affected patients (2, 6, 7). Furthermore, it was reported that cortisol autonomy is associated with an increased mortality (6, 8–10). Very recently, it was shown that affected female patients have a higher likelihood to die than women with NFA (2). As this effect was not detected in men, this study pointed to a clinically relevant sex disparity in patients with adrenal incidentalomas (2, 7).

This study aimed at comprehensively analysing the clinical course of patients with adrenal incidentalomas and cortisol autonomy. For this, the patient cohort from a large German tertiary referral centre specialized in adrenal disorders was stratified according to the guideline on the management of adrenal incidentalomas jointly published by the European Society of Endocrinology (ESE) and the European Network for the Study of Adrenal Tumours (ENSAT) in 2016 (1). We also focused on differences between certain sub-cohorts (e.g. uni- vs. bilateral tumours, conservative vs. surgical treatment, impact of changes in DST results over time).

## 2 Methods

### 2.1 Patient cohort

Adult patients with an adrenal incidentaloma who were referred to the University Hospital Würzburg between January 1^st^ 1998 and December 31^st^ 2017 were identified via chart review.

Patients were excluded for any of the following reasons: a) no confirmed adrenal incidentaloma (e.g. initial imaging was specifically performed because of a suspected adrenal mass); b) insufficient data on imaging and/or hormonal evaluation; c) overt adrenal hormone excess (e.g. Cushing’s syndrome, pheochromocytoma, or primary hyperaldosteronism); d) extra-adrenal malignancy already present at initial diagnosis of the adrenal incidentaloma; e) follow-up duration of < 24 months.

Adrenal lesions were classified as benign if a) adenomas or myelolipomas were confirmed histologically; b) tumours with non-malignant features were described radiologically, taking into account the criteria for interpretation of imaging procedures published in the ESE/ENSAT guideline (1); or c) patients had an inconspicuous follow-up of ≥ 60 months. If none of these criteria was fulfilled, patients were excluded from further analysis.

Written informed consent to either the ENSAT registry or the NeoExNet registry was obtained from all patients who were finally included into the current study. Both registries were approved by the Ethics committee of the University of Würzburg (88/11, 85/12).

As proposed in the ESE/ENSAT guideline (1), patients were classified into three study subgroups (using the serum cortisol level after the DST for stratification): ACS, >5.0 µg/dl; PACS, 1.9-5.0 µg/dl; NFA, ≤1.8 µg/dl. In patients who underwent a DST more than once during follow-up, the first test was used for stratification. If two or more tests from were available (that were carried out at least 12 months apart from each other), the development over time was assessed and classified as stable, improved or worsened (applying the above-defined categories).

Of note, 206 of the here reported 260 patients (79.2%) were also part of a recently published large international multi-centre study mainly focusing on mortality in patients with adrenal incidentalomas and cortisol autonomy (2). In contrast, our current study aims at providing country-specific information on the clinical, radiological, biochemical, and surgical course of affected patients. Accordingly, both study designs have a minor overlap only.

### 2.2 Follow-up

At initial diagnosis of the adrenal incidentaloma and at each follow-up visit, available retrospective data on living status, clinical examination, imaging procedures, biochemical analyses, cardiovascular morbidity, and potential therapies were collected. According to our clinical routine, an appointment in our endocrine outpatient clinic was proposed to those patients who were found to have insufficient follow-up according to the above defined criteria. In such cases, a standardized diagnostic workup (including clinical and laboratory evaluation) was performed. Patients with insufficient radiological or biochemical workup (according to the ESE/ENSAT guideline criteria (1)) were advised to undergo adequate diagnostic measures. Furthermore, standardized telephone interviews (with questionnaires on clinical status, medication, cardiovascular status, and potential adrenalectomy) were performed to complete and update data obtained during the retrospective chart review. A follow-up duration of at least 24 months was requested for inclusion.

### 2.3 Cardiovascular risk factors

The following cardiovascular risk factors were considered: arterial hypertension, diabetes mellitus, dyslipidemia, obesity, and smoking status. The presence of any of these risk factors was assumed if: a) corresponding diagnoses were listed in the patient’s chart; b) the diagnoses were reported during the telephone interview; c) a disease-specific therapy was taken; or d) clinical or biochemical assessment revealed clinically meaningful abnormalities.

### 2.4 Cardiovascular and thromboembolic events

Information on cardiovascular events was obtained from patients’ charts, follow-up visits in the outpatient clinic, or telephone interviews. The following cardiovascular (A) and thromboembolic (B) events were recorded: (A) myocardial infarction with or without ST elevation, coronary heart disease with previous interventions (including percutaneous coronary intervention, bypass surgery, and lysis), and stroke; (B) deep vein thrombosis and pulmonary embolism.

### 2.5 Statistical analysis

Statistical analysis was performed using IBM SPSS Statistics (Version 25.0) and GraphPad Prism (Version 9.3.0). At least ordinally scaled data are provided as median (interquartile range) and categorical data are provided as absolute numbers (percentage). For comparison of subcohorts, t-tests and ANOVA (for normally distributed data) or Mann-Whitney-U-Test and Kruskal-Wallis test (for non-parametric data) or chi-square (χ^2^) test (for dichotomous variables) were used. A two-sided P-value < 0.05 was considered significant. Separate incidence rates for cardiovascular and thromboembolic events are provided as hazard ratios (HR) along with 95% confidence intervals (CI). Kaplan-Meier plots were used to illustrate (incidence-free) survival, which was compared between groups by Log Rank-Test. Uni- and multivariate backward stepwise Cox regression analysis was used to identify predictors of cardiovascular and thromboembolic events (as outlined by HR and their corresponding 95% CI). During Cox regression analysis, the following variables were used as continuous variables: age (increase by 1 year), body mass index (BMI; increase by 1kg/m²), cortisol after DST (increase by 1µg/dl), glycated haemoglobin (HBA1c; increase by 1%), and total cholesterol (1mg/dl).

## 3 Results

### 3.1 Patient cohort

The initially identified patient cohort comprised 3854 patients. Applying the above-mentioned inclusion and exclusion criteria, 260 patients were finally included into the study. The detailed selection process of study candidates is provided in **Supplemental Figure 1**.

At initial diagnosis of the adrenal incidentaloma, 123 patients (47.3%) had NFA, while 96 patients (36.9%) had PACS and 41 patients (15.8%) had ACS. The majority of patients was female (n=147/260 (56.5%)), with comparable sex distribution between the three study subgroups. Overall, median age at initial diagnosis was 59.5 (20-82) years, and median follow-up was 8.8 (2.0-20.8) years. For each study subgroup, patient characteristics are displayed in **Table 1**. Details on smoking status are provided in **Supplemental Table 1**.

**Table 1:** Clinical characteristics of patients with adrenal incidentalomas (either non-functioning adenomas or adenomas with cortisol autonomy).

	NFA (n=123)	PACS (n=96)	ACS (n=41)	p-value
Demographics				
Sex, female *(n, %)*	69 (56.1%)	53 (55.2%)	25 (61.0%)	n.s. (0.818)
Age at initial diagnosis *(years)*	57 (62)	63 (48)**	56 (51)	<0.005
Follow-up *(years)*	8.8 (17.8)	8.5 (20.4)	9.8 (17.5)	n.s. (0.700)
Deceased *(n, %)*	8 (6.5%)	14 (14.6%)	11 (26.8%)***	<0.005
Imaging characteristics at initial diagnosis				
Left sided tumour *(n, %)*	68 (55.3%)	43 (44.8%)	21 (51.2%)	n.s. (0.419)
Bilaterality *(n, %)*	10 (8.1%)	21 (21.9%)*	12 (29.3%)***	<0.001
Largest tumour diameter *(mm)*	21.5 (75.0)	30.0 (106.0)***	31.0 (84.0)***	0.001
Biochemical testing at initial diagnosis				
ACTH *(ng/l)^1^*	12.2 (41.7)	11.4 (36.6)	9.3 (32.4)	n.s. (0.061)
Serum cortisol after 1-mg DST *(µg/dl)^2^*	1.3 (1.3)	2.7 (3.1)	8.1 (20.4)	<0.001
Late-night salivary cortisol *(µg/dl)^3^*	0.07 (1.23)	0.10 (1.01)	0.16 (1.53)	n.s. (0.076)
24-h urinary free cortisol *(µg/d)^4^*	53.8 (403.4)	47.6 (271.1)	52.0 (342.6)	n.s. (0.848)
Therapeutic approach				
Conservative	101 (82.1%)	73 (76.0%)	25 (61.0%)**	<0.05
Surgical	22 (17.9%)	23 (24.0%)	16 (39.0%)**	<0.05

Values are absolute (percentage) or median (range) if not stated otherwise. According to the serum cortisol level after the first 1 mg dexamethasone suppression test, patients were stratified into the three study subgroups NFA, PACS, and ACS. As data were not available from all patients, the respective cohort size is outlined: ^1^, n=220; ^2^, 23/260 (8.8%) patients had an initial DST with 2 or 3 mg dexamethasone; ^3^, n=119; ^4^, n=180. Significance is provided according to univariate ANOVA analysis, comparing all three study subgroups. Comparisons of study subgroups amongst each other are annotated as asterisks: *, P<0.05; **, P<0.01; ***, P<0.001 (in each case compared to NFA).

ACS, autonomous cortisol secretion; ACTH, adrenocorticotropic hormone; DST, dexamethasone suppression test; NFA, non-functioning adenoma; n.s., not significant; PACS, possible autonomous cortisol secretion.

### 3.2 Diagnostic workup

#### 3.2.1 Radiological course

In total, 878 imaging procedures were documented, leading to a median number of 4 (1–13) imaging procedures per patient (computed tomography (CT), n=491; magnetic resonance imaging (MRI), n=173; ultrasound, n=134; position emission tomography (PET) along with CT, using flourodesoxyglucose as tracer (FDG-PET/CT), n=49). In 201 patients, at least one follow-up imaging was available (with a median interval of 25 (0-206) months between the first and the last procedure). Median largest tumour diameters in the entire patient population were 27.0 (10-116) mm at initial diagnosis and 27.0 (9-116) mm at last follow-up. According to the original radiological reports, an increase of the largest tumour diameter of ≥20% was observed in 24 (11.9%) of these cases during a median follow-up of 25 months (0-206) (rates per subgroup: NFA, 12.1%; PACS, 12.3%; ACS, 10.3%). In 12 of these cases, a follow-up DST was available, demonstrating a shift from NFA to ACS in 3 of them (25%), while the hormonal secretion pattern remained stable in the other patients (NFA, n=3; PACS, n=5 ACS, n=1).

#### 3.2.2 Hormonal course

In total, 479 DST were performed in 260 patients (median 1 (1–11) DST per patient). As patients were stratified according to their DST results, the serum cortisol levels after DST differed significantly between the three study subgroups, while the time-span between first and last DST as well as the individual number of DST were comparable. Results of additional biochemical testing performed in a subset of patients at initial diagnosis are provided in **Table 1**.

At least two DST were performed in 124 (47.7%) patients (with only pre-operative DST being counted in case of surgery during follow-up). The median time interval between the first and the last DST was 41 (3-214) months. Eighty-four (67.7%) patients demonstrated a stable cortisol secretion pattern. While 21 (16.9%) patients shifted toward a worse DST category (e.g. from NFA to PACS or ACS), 19 (15.3%) patients demonstrated an improvement (**Table 2**; **Supplemental Figure 2**).

**Table 2:** Outcome changes of the dexamethasone suppression test during follow-up.

Stratification according to the last DST	Stratification according to the DST at initial diagnosis		
	NFA (n=53)	PACS (n=47)	ACS (n=24)
NFA	stable NFA 71.7%	improved 17.0%	improved 0%
PACS	worsened 20.8%	stable PACS 70.2%	improved 45.8%
ACS	worsened 7.5%	worsened 12.8%	stable ACS 54.2%

ACS, autonomous cortisol secretion; DST, dexamethasone suppression test; NFA, non-functioning adenoma; PACS, possible autonomous cortisol secretion.Color codes are as follows: green, improved diagnostic outcome according to the last DST; yellow, similar diagnostic outcome of both DST; red: worsened diagnostic outcome according to the last DST.

#### 3.2.3 Clinical course

**Figure 1:**
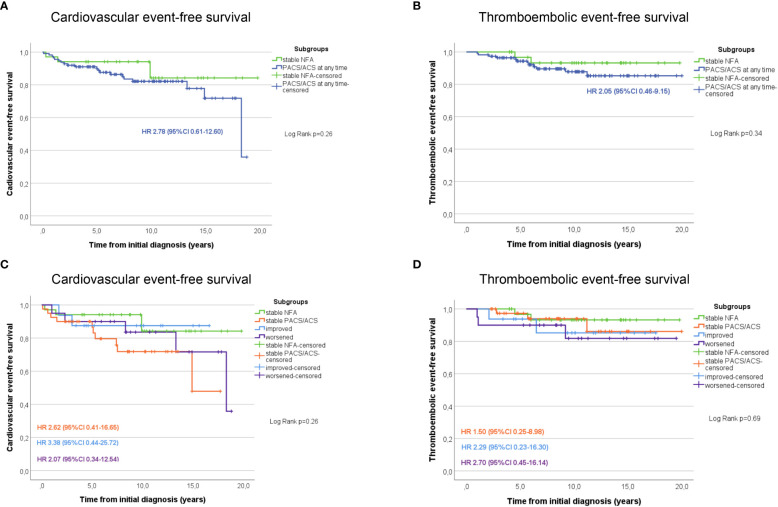
Kaplan Meier estimates of cardiovascular **(A)** and thromboembolic **(B)** event-free survival in patients with NFA compared to those with PACS or ACS at any time point during follow-up. Additional graphs illustrate the cardiovascular **(C)** and thromboembolic **(D)** event-free survival in patients stratified according to the outcome changes of the dexamethasone suppression test. ACS, autonomous cortisol secretion; CI, confidence interval; HR, Hazard-ratio; NFA, non-functioning adenoma; PACS, possible autonomous cortisol secretion.

Non-operated patients with stable NFA had the lowest rates of arterial hypertension, diabetes mellitus, dyslipidemia, and obesity compared to patients with stable PACS or ACS on the one hand, and to patients in whom DST category changed over time on the other (**Supplemental Table 2**). During follow-up, non-operated patients with stable NFA had the lowest cumulative risk for cardiovascular and thromboembolic events of all study subgroups (**Figure 1**). Of note, no patient of the entire cohort developed a clinically overt Cushing’s syndrome during follow-up.

### 3.3 Therapeutic outcome

While 199 (76.5%) patients were followed-up conservatively, the other 61 (23.5%) patients underwent adrenalectomy. Both therapeutic modalities were analysed separately and compared among each other regarding cardiovascular risk profile and outcome.

#### 3.3.1 Conservatively treated patients

**Figure 2:**
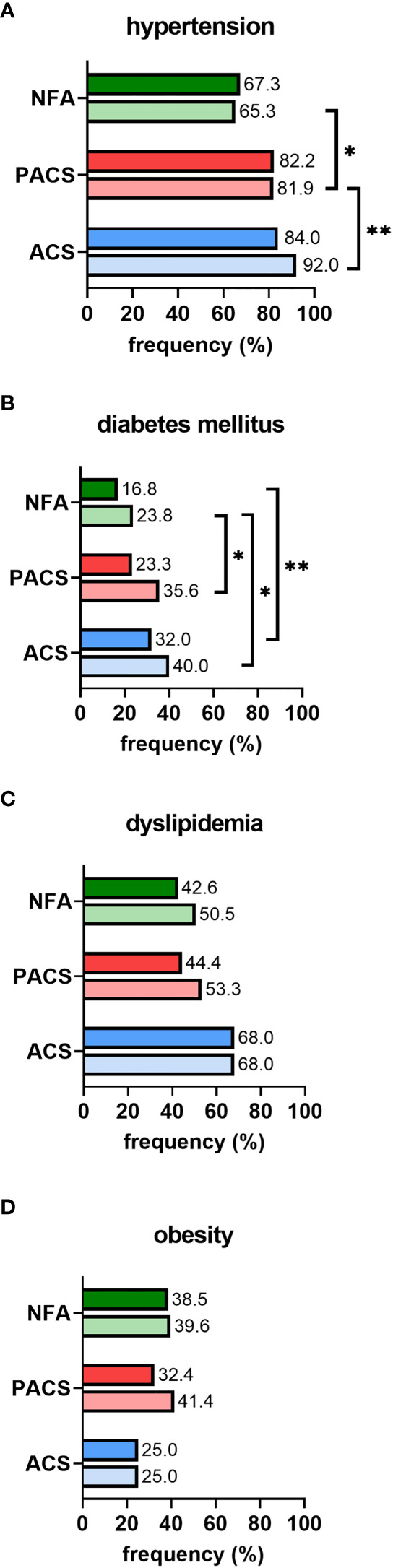
Prevalence of cardiovascular risk factors in conservatively treated patients with adrenal incidentalomas: **(A)** hypertension, **(B)** diabetes mellitus, **(C)** dyslipidemia, **(D)** obesity. According to the first 1-mg dexamethasone suppression test result, patients were stratified into the three study subgroups NFA (n=101), PACS (n=73), and ACS (n=25). Prevalence is separately reported for initial diagnosis (upper full bar) and last follow-up (lower shaded bar). Comparisons of study subgroups amongst each other are annotated as asterisks: *p<0.05; **p<0.01. ACS, autonomous cortisol secretion; NFA, non-functioning adenoma; PACS, possible autonomous cortisol secretion.

In non-operated patients, cardiovascular risk factors became more prevalent over time, as illustrated by increasing frequencies of affected patients from initial diagnosis to last follow-up (**Figure 2**). Furthermore, except for obesity, the prevalence of arterial hypertension, diabetes mellitus, and dyslipidemia increased with post-DST serum cortisol levels. Intake of antihypertensive drugs was less prevalent in NFA than in PACS and ACS, and this was true not only at baseline (63.3% vs. 65.8% and 72.0%; p<0.001) but also at last follow-up (57.4% vs. 80.3% and 87.0%; p<0.001). At final follow-up, patients with NFA also took significantly less antihypertensive drugs than ACS patients (2 (1-4) vs. 3 (1-8); p<0.01). In contrast, no significant differences in the rate of patients under antidiabetic and lipid-lowering treatment (and the number of associated drugs) were observed between the three study subgroups (data not shown).

**Figure 3:**
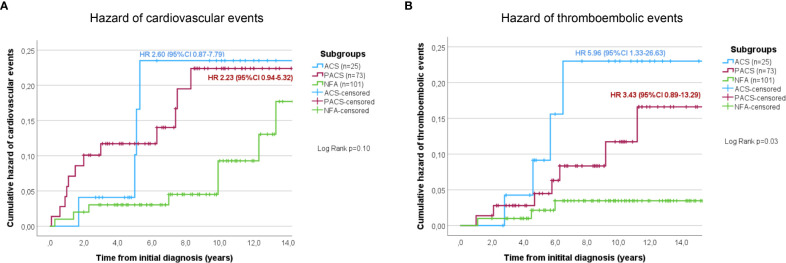
Cumulative hazard of cardiovascular **(A)** and thromboembolic **(B)** events in conservatively treated patients with adrenal incidentalomas. According to the initial 1-mg dexamethasone suppression test result patients were stratified into the three study subgroups NFA, PACS, and ACS. ACS, autonomous cortisol secretion; NFA, non-functioning adenoma; PACS, possible autonomous cortisol secretion.

At initial diagnosis, previous cardiovascular events were slightly more often reported in patients with cortisol autonomy, but the respective rates did not differ significantly between the three study groups (NFA, 8.9%; PACS, 17.8%; ACS, 16.0%; p=0.20, χ^2^ = 3.17). If previous thromboembolic events were considered, no significant differences among the three groups were observed (NFA, 5.0%; PACS, 6.8%; ACS, 12.0%; p=0.44, χ^2 ^= 1.65). After initial diagnosis of the adrenal incidentaloma, 31 cardiovascular events (cardiac, n=25; cerebrovascular, n=6) and 17 thromboembolic events were reported in 27 (13.6%) and 14 (7.0%) non-operated patients, respectively. The frequency of cardiovascular events slightly increased by DST category (NFA, 10.9%; PACS, 20.5%; ACS, 20.0%; p=0.18, χ^2^ = 3.43). **Figure 3A** illustrates a trend towards an increasing risk of cardiovascular events in PACS (HR 2.23, 95%-CI 0.94-5.32; p=0.09) and ACS (HR 2.60, 95%-CI 0.87-7.79; p=0.07) compared to NFA. Univariate Cox regression analysis indicated former cardiovascular events (p<0.001), higher cholesterol levels (p=0.007), and sex (p=0.02) as significant predictors of cardiovascular events, whereas increasing levels of post-DST serum cortisol were only associated with a trend (p=0.05). Of these, only previous cardiovascular events (HR 3.78, 95%-CI 1.17-12.22; p=0.03) and sex (HR 0.31, 95%-CI 0.09-0.99; p=0.049) remained significant predictors after multivariate Cox-regression analysis. The frequency of thromboembolic events significantly increased by DST category (NFA, 4.0%; PACS, 10.9%; ACS, 20.0%; p=0.02, χ^2^ = 7.46). Compared to NFA, patients with PACS (HR 5.96, 95%-CI 1.33-26.63; p=0.02) and ACS (HR 3.43, 95%-CI 0.89-13.29; p=0.07) showed a higher risk for thromboembolic events (**Figure 3B**). Univariate Cox regression analysis revealed that increasing levels of post-DST serum cortisol (p=0.005), Hb1Ac (p=0.02), and BMI (p=0.02) were significantly correlated with thromboembolic events, whereas a trend was observed for increasing age (p=0.75). After multivariate analysis, only increasing BMI remained a significant predictors of thromboembolic events (HR 1.23, 95%-CI 1.00-1.51; p=0.046), while a trend was observed for increasing levels of post-DST serum cortisol (HR 1.16, 95%-CI 0.99-1.35; p=0.06).

Twenty-five (12.6%) of the conservatively treated patients deceased during follow-up. Mortality-rates differed significantly between the three study subgroups (NFA, 6.9%; PACS, 15.1%; ACS, 28.0%; p<0.05), with a significantly increased mortality risk in ACS patients compared to NFA (HR 4.7, 95%-CI 1.6-13.3; p<0.005). Cause of death could be assessed in 17 (68.0%) patients, with the majority being related to malignancies (n=8, 47.1%) followed by cardiovascular (n=6, 35.3%) or thromboembolic events (n=1, 5.9%), and infections (n=2, 11.8%). Taken together, cardiovascular and thromboembolic mortality was significantly higher in ACS than in NFA (HR 14.6, 95%-CI 1.5-140.7; p<0.05). Lethal infections occurred in each one case with NFA and ACS.

#### 3.3.2 Surgically treated patients

Surgical treatment was significantly less frequently performed in NFA compared to PACS and ACS (rates of operated patients per subgroup: 17.9% vs. 24.0% and 39.0%; see **Table 1**).

With respect to the entire population of 61 operated patients, radiological findings suspicious of malignancy followed by hormonal activity were the most frequent indications for surgery (**Table 3**). Median time from initial diagnosis to surgery was 5 (0-148) months, and median postoperative follow-up was 72 (6-214) months. Most patients underwent laparoscopic surgery (n=43, 71.7%). According to the pathological reports, 51 (83.6%) of the resected lesions were adenomas and 10 (16.4%) were myelolipomas. Both tumour entities did not significantly differ regarding tumour size and distribution across the three study subgroups.

**Table 3:** Clinical characteristics of patients with adrenal incidentalomas (either non-functioning adenomas or adenomas with cortisol autonomy) undergoing adrenal surgery.

	NFA (n=22)	PACS (n=23)	ACS (n=16)	p-value
Demographics				
Sex, female *(n, %)*	12 (54.5%)	16 (69.6%)	10 (62.5%)	n.s. (0.596)
Age at initial diagnosis *(years)*	52.5 (52)	59 (47)	50 (42)	n.s. (0.059)
Time from initial diagnosis to surgery *(months)*	5 (147)	9 (144)	5 (18)	n.s. (0.128)
Time from surgery to last follow-up *(months)*	56 (104)	61 (208)	114 (190)*	<0.05
Tumour characteristics				
Left sided tumour *(n, %)*	11 (57.9%)	9 (42.9%)	8 (72.7%)	n.s. (0.815)
Unilateral localization *(n, %)*	19 (86.4%)	21 (91.3%)	11 (68.8%)	n.s. (0.097)
Tumour diameter at initial diagnosis *(mm)*	39 (16-80)	41 (10-75)	40 (27-94)	n.s. (0.850)
Indication for surgery ^1^				
Suspicion of malignancy *(n, %)*	15 (68.2%)	11 (50.0%)	4 (25.0%)	<0.05
Hormonal activity *(n, %)*	2 (9.1%)	3 (13.6%)	9 (56.3%)	<0.005
Suspicion of malignancy and hormonal activity *(n, %)*	0 (0%)	2 (9.1%)	2 (12.5%)	n.s. (0.275)
Patients’ desire *(n, %)*	1 (4.5%)	1 (4.5%)	1 (6.3%)	n.s. (0.966)
Other	0 (0%)	3 ^a^ (13.6%)	0 (0%)	n.s. (0.066)
Details on the surgical intervention				
Minimally invasive surgery *(n, %)*	15 (86.4%)	17 (89.5%)	11 (91.7%)	n.s. (0.539)
Duration of inpatient hospital stay *(days)*	6 (12)	5 (20)	5.5 (10)	n.s. (0.465)
Complications *(n, %) ^2^*	6 (30.0%)	4 (20.0%)	1 (7.1%)	n.s. (0.275)
Adrenal insufficiency				
Postoperative adrenal insufficiency *(n, %) ^3^*	1 ^b^ (4.5%)	9 (42.9%)**	9 (64.3%)**	<0.001
Duration of adrenal insufficiency *(months)*	47 (-)	4.5 (40)	17 (130)	n.s. (0.310)

Values are absolute (percentage) or median (interquartile range) if not stated otherwise. Patients were stratified into the three study subgroups NFA, PACS, and ACS according to the serum cortisol level after the first 1-mg dexamethasone suppression test. If data was not available from all patients, the respective cohort size is provided: ^1^, n=54; ^2^, n=54; ^3^, n=57, other indications for surgery were the inclusion into a clinical trial, the development of an adrenal bleeding, and the presence of a concomitant renal cell carcinoma. ^b^, operated due to shift from NFA to ACS over time (with persistent postoperative adrenal insufficiency). Significance is provided according to univariate ANOVA analysis, comparing all three study subgroups. Comparisons of study subgroups amongst each other are annotated as asterisks: *, p<0.01; **, p<0.005 (in each case compared to NFA).

ACS, autonomous cortisol secretion; ACTH, adrenocorticotropic hormone; DST, dexamethasone suppression test; NFA, non-functioning adenoma; n.s., not significant; PACS, possible autonomous cortisol secretion.

Overall, the median stay in hospital was 5 (2-24) days. In 11 out of 54 (20.4%) patients with available information, a broad spectrum of mostly low grade postoperative complications was reported (**Supplemental Table 3**). If adrenal insufficiency was assumed, an adrenocorticotropin stimulation test was performed. Adrenal insufficiency was observed in 19 out of 56 (33.9%) evaluable patients. As outlined in **Table 3**, postoperative adrenal insufficiency was - as expected - more prevalent in patients with PACS and ACS. Of note, a single patient with NFA underwent surgery due to a shift towards ACS over time and suffered from persistent adrenal insufficiency postoperatively.

The prevalence of arterial hypertension decreased significantly from initial diagnosis to last post-operative follow-up (77.0% vs. 61.7%; p<0.05), while the pre- and postoperative rates of patients with diabetes mellitus, dyslipidemia and obesity remained comparable. Eight (13.1%) of the operated patients deceased during follow-up. Cause of death could be assessed in five (62.5%) patients, with three being of infectious and two of cardiovascular nature.

#### 3.3.3 Comparison of conservatively and surgically treated patients

Conservatively treated patients had significantly lower post-DST serum cortisol levels (1.8 (0.5-21.5) vs. 2.45 (0.5-25.5) µg/dl; p<0.05) and were significantly older (61 (20-80) vs. 53 (23-78) years; p<0.01) than surgically treated patients, whereas no significant difference in sex distribution, frequency of bilateral tumours, and time of follow-up were observed.

At baseline, cardiovascular risk factors between conservatively and surgically treated patients were comparable. At last follow-up, however, surgically treated patients had a lower prevalence of all assessed risk factors, with significantly less arterial hypertension (61.7% vs. 74.9%; p<0.05) (**Supplemental Table 4**).

When conservatively and surgically treated patients were compared amongst each other, the rates of those with cardiovascular events occurring either before (n=26 (13.1%) vs. n=9 (14.8%); p=0.73) or after (n=27 (13.6%) vs. n=8 (13.1%); p=0.93) the initial diagnosis of the adrenal incidentaloma were not significantly different. Former thromboembolic events were also reported in similar frequency (n=13 (6.5%) vs. n=4 (6.6%); p=0.99), whereas thromboembolic events occurring during follow-up were only observed in conservatively treated patients and not in those undergoing surgery (n=14 (7%) vs. n=0 (0%); p=0.03, χ^2^ = 4.54). Moreover, mortality rates were comparable between operated and non-operated patients (13.1% vs. 12.6%; n.s.). A similar observation was made when operated and non-operated patients were compared within the three study subgroups (data not shown).

### 3.4 Patients with bilateral adrenal adenomas

At initial diagnosis of the adrenal incidentaloma, 43 patients (16.5%) had bilateral lesions, and this rate slightly increased to 47 patients (18.1%) until last follow-up. Patients with bilateral adenomas did not significantly differ from those with unilateral adenomas regarding sex, age at initial diagnosis, duration of follow-up, and rates of adrenalectomies (data not shown). However, bilateral disease was associated with significantly larger tumours (33 (16-106) vs. 25 (9-116) mm; p<0.01) and more hormonal activity (as outlined by a higher prevalence of bilateral tumours in PACS and ACS compared to NFA (**Table 1**)). Besides, post-DST serum cortisol levels were significantly higher in bilateral than in unilateral tumours (3.5 (0.5-21.5) vs. 1.8 (0.5-25.5) µg/dl; p<0.001)). At follow-up, a tendency towards a higher prevalence of cardiovascular risk factors in bilateral compared to unilateral tumours was found, which was significant for arterial hypertension (87.0% vs. 68.4; p<0.05) but not for diabetes mellitus (40.4% vs. 26.8%), dyslipidemia (51.5% vs. 51.6%), and obesity (40.0% vs. 37.3%). During follow-up, frequencies of cardiovascular events (15.2% vs. 20.9%; p=0.93) and thromboembolic events (6.4% vs. 6.9%; p=0.90) were similar between patients with unilateral and bilateral disease. Finally, mortality rates were also comparable between patients with bilateral (n=6/47, 12.8%) and unilateral (n=27/213, 12.7%) disease (n.s.). In 9 cases (20.9%), bilateral adrenalectomy was performed.

## 4 Discussion

Here, we report the first German cohort of well-characterized patients with adrenal incidentaloma and cortisol autonomy. We confirmed an increasing prevalence of relevant cardiovascular risk factors (i.e., arterial hypertension, dyslipidemia, and diabetes mellitus), cardiovascular and thromboembolic events, and mortality with rising degree of cortisol autonomy. Adrenalectomy resulted in a significantly lower prevalence of arterial hypertension and thromboembolic events (in contrast, only a tendency towards a lower risk was observed for cardiovascular events). Patients suffering from bilateral disease had higher cortisol levels and a higher prevalence of cardiovascular risk factors, while the frequency of cardiovascular events, thromboembolic events, and mortality were comparable to patients with unilateral disease. Of note, more than 30% of patients required reclassification if consecutive DST were performed.

Over the last few years, an increasing number of studies (2, 6–10) reported on cardiovascular morbidity and mortality in patients with adrenal incidentalomas stratified according to the diagnostic criteria given in the ESE/ENSAT guideline from 2016 (1). With NFA constituting roughly half of our patients (47.3%), we here report a comparable distribution of patients across the three study subgroups (despite a slightly higher percentage of cases with ACS (15.8%)). Furthermore, a similar pattern of demographic data (e.g. age and sex) and tumour characteristics (e.g. size and localization) make this German cohort well comparable to previous studies with less preselected patients from other (mostly European) countries (2, 6–10).

We observed a higher prevalence of arterial hypertension, diabetes mellitus, and dyslipidemia in PACS and ACS than in NFA (of note, the same pattern was found both at initial diagnosis and at last follow-up). Although this finding is generally in good accordance with a large meta-analysis (11), we detected even higher prevalence rates. This discrepancy is probably best explained by local differences regarding cardiovascular risk profiles and definitions of cardiovascular risk factors. Accordingly, country-specific reference data should ideally be aimed for.

Furthermore, we found a slightly increased rate of cardiovascular events and a significantly increased rate of thromboembolic events in patients with cortisol autonomy compared to those with NFA. Even though definitions of cardiovascular events differed slightly, comparable results were reported for two cohorts from Italy (8) and Sweden (10) and a large international multicentre study published very recently (2). Accordingly, these observations would underline the ESE/ENSAT guideline recommendation that at least patients with small non-functioning adrenal adenomas of less than 4 cm do not require any further follow-up (1). However, when the analysis was restricted to patients with consecutive DST results, the lowest risk for cardiovascular events was observed in case of stable NFA, while a worsened biochemical profile came along with the highest risk. As a result, it is our impression that recommendations for further follow-up in patients with NFA should not be too restrictive (as those with worsened secretion pattern over time may benefit from endocrine reevaluation as well).

Interestingly, patients with bilateral adrenal masses had a slightly higher prevalence of cardiovascular risk factors than those with unilateral incidentalomas. However, frequency of cardiovascular events, thromboembolic events, and mortality rates were comparable. Previous studies described similar results for cardiovascular risk factors (12, 13), whereas cardiovascular events, thromboembolic events, and mortality were not assessed.

The ESE/ENSAT guideline recommends conservative treatment for most patients with PACS and ACS, thereby particularly aiming at optimal treatment of comorbidities (1). Of note, however, the present study indicates a favourable outcome of surgical treatment in such patients. This is well in line with a large meta-analysis (with 26 studies) (11) and a review (with 8 studies) (14). Nevertheless, it has to be pointed out that only one of the investigated studies had a prospective design. In this randomized controlled trial with 23 patients, an improvement of arterial hypertension (in 12/18, 67%), glucose metabolism (in 5/8, 63%), and body weight (in 3/6, 50%) was reported (15). Very recently, another prospective randomized-controlled trial (the first that applied the stratification system recommended in the ESE/ENSAT guideline from 2016) was also able to demonstrate a significant improvement of arterial hypertension and glycometabolic control in surgically compared to conservatively treated patients with PACS (16). In this study, 68% of operated and 13% of non-operated patients (p=0.001) experienced improvement of blood pressure at follow-up; glycometabolic control improved in 28% and 3%, respectively (p=0.02). Results of further prospective randomized-controlled trials (NCT02364089; NCT04860180; NCT01246739) are currently pending, hopefully paving the way for clear treatment recommendations.

Despite the fact that evidence for benefits from surgery on the cardiovascular risk profile is emerging, none of the above-mentioned studies assessed mortality as primary end-point. However, this was the case in a recent large retrospective international multi-centre study including 3656 patients with adrenal incidentalomas (2). Here, all-cause mortality was found to be particularly elevated in women with ACS younger than 65 years. In contrast, no significant difference in mortality rates was observed in men if the three study subgroups were compared amongst each other. Due to its retrospective design, this study could certainly not establish a causative relationship between the degree of cortisol autonomy and mortality (but still provides reasonable evidence for a strong association). Large-scale prospective studies with a long-term follow-up are needed to reliably assess mortality as end-point.

In our study, operated patients most frequently underwent laparoscopic adrenalectomy (84.3%). However, the rate of perioperative complications (20.4%) was higher than previously reported for this surgical approach (15, 17, 18). Possible explanations are different definitions of complications in our study compared to the former literature and a referral bias to our centre (which represents a reference centre for adrenal diseases and may therefore oversees a series of more complex cases). The latter explanation is supported by the fact that in our cohort operated tumours were larger than in other studies (median tumour diameter of 45 mm vs. 29-34 mm) (15, 19, 20).

In our present cohort, no patient developed overt Cushing’s syndrome. This is well in line with other studies that described an underlying risk of less than 0.5% (1, 2, 21, 22). Of note, differences between PACS and ACS on the one hand and cortisol-producing adrenal adenomas associated with overt Cushing’s syndrome on the other have been reported both at the genetic and at the transcriptome level (23–25). Most cortisol-producing adenomas with overt Cushing’s syndrome are associated with mutations in the cAMP/PKA pathway (*PRKACA* and *GNAS*), which are less frequency detected in patients with PACS or ACS (23–27). Moreover, transcriptome profiles from patients with PACS and ACS showed more similarities with NFA than with tumours associated with overt Cushing’s syndrome, and this observation was independent from the genetic background (24).

With 67.7%, the majority of our patients who underwent more than one DST demonstrated stable cortisol secretion throughout a median follow-up of 41 months. Interestingly, though, 28.3% of our patients initially classified as NFA developed PACS or ACS. In contrast, a recent meta-analysis reported a progression rate of only 4.3% (22). However, the majority of studies that were included into this meta-analysis did not apply the specific diagnostic criteria recommended in the ESE/ENSAT guideline (1). Accordingly, our current finding is in better accordance with studies where application of the same diagnostic criteria resulted in progression rates of up to 31.4% (8, 10). This fact would support the call for follow-up DST particularly in patients initially diagnosed as NFA (28, 29). Nevertheless, we also observed an improvement from initial PACS or ACS towards NFA in 8 out of 71 patients (11.3%), which is slightly higher than previously reported rates of 10% or less (22, 28). As a result, prospective long-term studies with large cohorts and centralized analytical workup are required to estimate the true conversion rates of post-DST results over time.

Limitations of the present study include the retrospective monocentric design that might have led to selection, information, and referral bias. Furthermore, data have been acquired from a mostly Caucasian population. Hence, generalizability of results might be limited. Moreover, the required follow-up interval of at least two years (which was chosen to allow for a clear association between cortisol autonomy and e.g. cardiovascular morbidity) is arbitrary and may have excluded some patients with shorter development of relevant comorbidities or events. On the other hand, strengths of our study include the large and clinically well-defined patient cohort and the long follow-up compared to previous studies (10, 30). Furthermore, the comprehensive assessment and comparison of different sub-cohorts make this study stand out from the available literature on adrenal incidentalomas and cortisol autonomy.

In summary, this study confirms increased cardiovascular morbidity and mortality in patients with adrenal incidentalomas and cortisol autonomy. These patients should therefore be monitored carefully, including adequate treatment of cardiovascular risk factors. Adrenalectomy was associated with a significantly decreased prevalence of hypertension and thromboembolic events. However, prospective randomized controlled trials are needed to evaluate the clinical benefits of surgical treatment in patients with cortisol autonomy (not only with respect to cardiovascular risk factors but also with respect to cardiovascular events and overall mortality). In addition, more than 30% of patients required reclassification according to repeated dexamethasone suppression tests. Thus, cortisol autonomy should ideally be confirmed before making any relevant treatment decision (e.g. adrenalectomy).

## Data availability statement

The raw data supporting the conclusions of this article will be made available by the authors, without undue reservation.

## Ethics statement

The studies involving human participants were reviewed and approved by the Ethics committee of the University of Würzburg. The patients provided written informed consent to participate in this study.

## Author contributions

TD and MF designed the research. HR, SK, SM, BA, MD, and TD performed the statistical analyses. HR and TD drafted the manuscript. All authors collected samples and clinical data from patients, contributed to writing the manuscript, and approved the final version to be published. All authors contributed to the article and approved the submitted version.
