# Supplementary material for: Clinical course of patients with adrenal incidentalomas and cortisol autonomy: a German retrospective single center cohort study
Source: Front Endocrinol (Lausanne). 2023 May 8;14:1123132. doi: 10.3389/fendo.2023.1123132 (PMC10200872; doi:10.3389/fendo.2023.1123132)
Supplement: Supplementary file 1 [file DataSheet_1.docx]

Supplementary Material

Clinical course of patients with adrenal incidentalomas and cortisol autonomy – a German retrospective single center cohort study.

**Remde H^1^, Kranz S^1,2^, Morell S^1,3^, Altieri B^1^, Kroiss M^1,4^, Detomas M^1^, Fassnacht M^1^, Deutschbein T*^1,5^**

*** Correspondence:**

PD Dr. Timo Deutschbein

Department of Internal Medicine I,

Division of Endocrinology and Diabetes, University Hospital,

University of Würzburg,

Würzburg 97080, Germany

[Deutschbein_t@ukw.de](mailto:Deutschbein_t@ukw.de)

# Supplementary Methods

Minimal dataset requested from each patient

- Medical history: date of birth, date of initial diagnosis of the adrenal incidentaloma (including bodyweight and size at this time point), information on previous cardiovascular events.
- Clinical examination: presence of cardiovascular risk factors (i.e., obesity, arterial hypertension, diabetes mellitus, and dyslipidemia) and intake of associated medication at initial diagnosis of the adrenal incidentaloma.
- Laboratory data: serum-cortisol level after 1-mg dexamethasone suppression test.
- Imaging: radiographic images (e.g. abdominal computed tomography) and written radiological reports (including size of the adrenal incidentaloma at first diagnosis and last imaging).
- Clinical course: information on cardiovascular events occurring after the initial diagnosis of the incidentaloma and respective date.
- Last follow-up: date and cause of death or date of last follow-up with respective clinical information (presence of obesity, arterial hypertension, diabetes mellitus, dyslipidemia and respective medication).

# Supplementary Figures


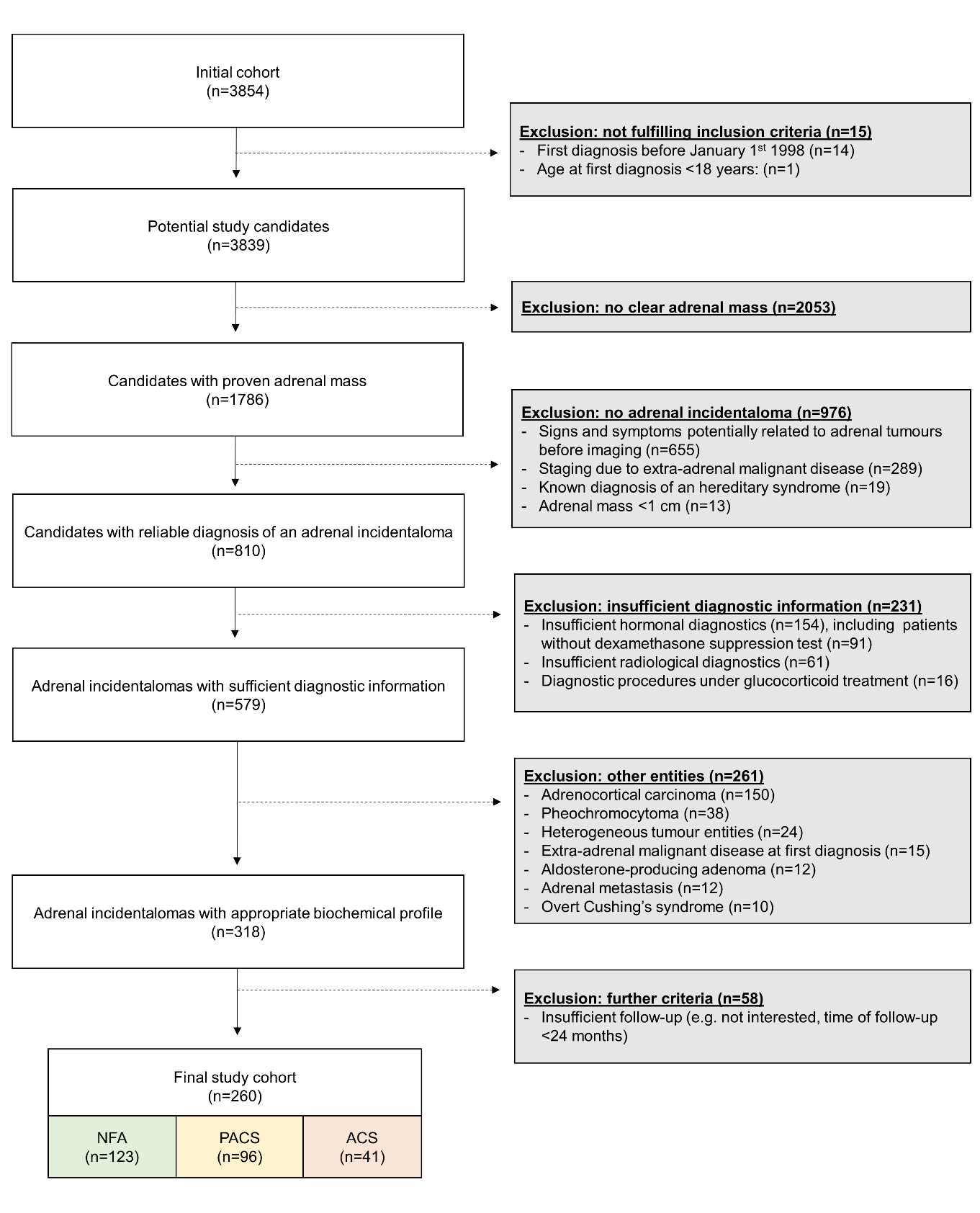


**Supplemental Figure 1**: Flow-chart of the selection process of the study cohort.

Abbreviations: ACS, autonomous cortisol secretion; NFA, non-functioning adenoma; PACS, possible autonomous cortisol secretion.


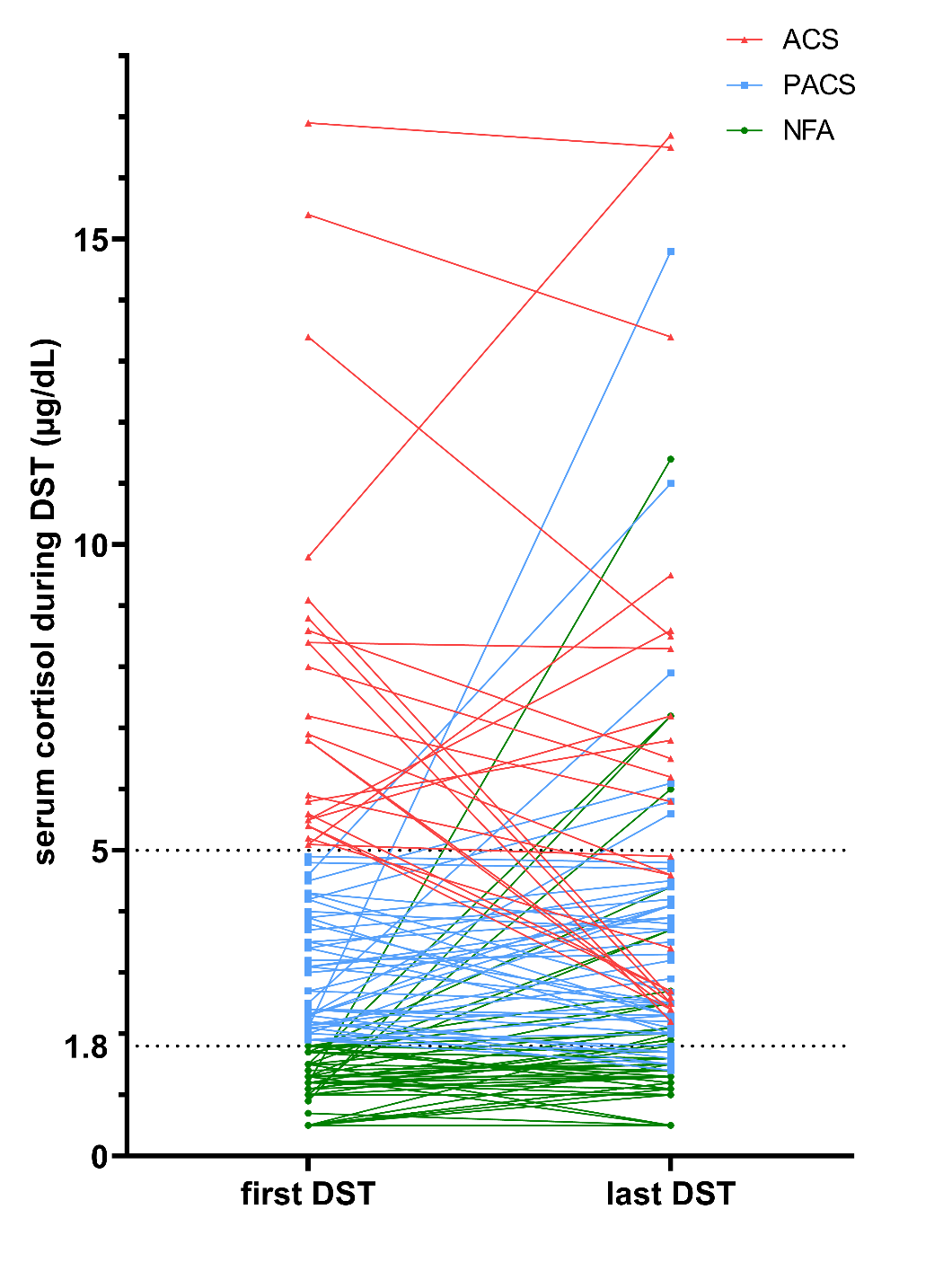


**Supplemental Figure 2:** Individual course of serum cortisol from the first to the last DST in patients with at least two available DST results (n=124). In case of operated patients, the last DST is the last test before surgery.

Abbreviations: ACS, autonomous cortisol secretion; DST, dexamethasone suppression test NFA, non-functioning adenoma; PACS, possible autonomous cortisol secretion.

# Supplemental Tables

**Supplemental Table 1:** Smoking status in patients with adrenal incidentalomas.

|  | NFA  (n=122) | PACS  (n=87) | ACS  (n=37) | P-value |
| --- | --- | --- | --- | --- |
| Never smoker (n,%) | 72 (59.0%) | 37 (42.5%) | 17 (45.9%) | <0.01 |
| Former smoker (n, %) | 19 (15.6%) | 26 (29.9%) | 8 (21.6%) | n.s. (0.104) |
| Current smoker (n,%) | 31 (25.4%) | 24 (27.6%) | 12 (32.4%) | n.s. (0.699) |
| Pack years ^1^ | 18 (3-80) | 20 (5-80) | 18 (5-80) | n.s. (0.581) |

In all patients, smoking status was assessed at the time of the last follow-up. ^1^ Data availability: n=115/120

Abbreviations: ACS, autonomous cortisol secretion; NFA, non-functioning adenoma; n.s., not significant; PACS, possible autonomous cortisol secretion.

**Supplemental Table 2:** Prevalence of cardiovascular risk factors in non-operated patients at last follow-up according to changes in the cortisol secretion pattern over time.

|  | Stable NFA | Stable PACS and ACS | Improved  biochemical  profile | Worsened  biochemical  profile | P-value |
| --- | --- | --- | --- | --- | --- |
| Arterial hypertension (n, %) | 17 (50.0 %) | 35 (87.5%) | 14 (87.5%) | 19 (95.0%) | <0.001 |
| Diabetes mellitus  (n, %) | 7 (20.6%) | 14 (35.0%) | 8 (50.0%) | 8 (40.0%) | n.s. (0.133) |
| Dyslipidemia  (n, %) | 19 (55.9%) | 24 (60.0 %) | 11 (68.8%) | 13 (65.0%) | n.s. (0.819) |
| Obesity  (n, %) | 10 (29.4%) | 18 (47.4%) | 6 (37.5%) | 7 (35.0%) | n.s. (0.465) |

Abbreviations: ACS, autonomous cortisol secretion; NFA, non-functioning adenoma; n.s., not significant; PACS, possible autonomous cortisol secretion.

**Supplemental Table 3**: Complications related to adrenalectomy.

| Complication | Total number | Grade I | Grade II | Grade III | Grade IVa |
| --- | --- | --- | --- | --- | --- |
| Acute pancreatitis | 1 | 0 | 0 | 0 | 1 |
| Allergic reaction | 2 | 1 | 1 | 0 | 0 |
| Hypokalemia | 1 | 1 | 0 | 0 | 0 |
| Hypoxia | 1 | 0 | 1 | 0 | 0 |
| Intestinal atony | 1 | 0 | 1 | 0 | 0 |
| Large hematoma | 1 | 1 | 0 | 0 | 0 |
| Lymph fistula | 2 | 1 | 1 | 0 | 0 |
| Pneumonia | 1 | 0 | 1 | 0 | 0 |
| Wound infection | 1 | 0 | 1 | 0 | 0 |

Postoperative complications were graded according to the Clavien-Dindo Classification (1). In addition, three patients experienced a pneumothorax during surgery. All three cases were treated by pleural suture, and an additional thoracic drainage was applied in two cases.

**Supplemental Table 4**: Prevalence of cardiovascular risk factors in conservatively and surgically treated patients at initial diagnosis of the adrenal incidentaloma and last follow-up.

|  | Conservatively treated patients (n=199) | Surgically treated patients (n=61) | P-value |
| --- | --- | --- | --- |
| Initial diagnosis | | | |
| Arterial hypertension (n, %) | 149 (74.9%) | 47 (77.0%) | n.s. (0.731) |
| Diabetes mellitus (n, %) | 42 (21.1%) | 11 (18.0%) | n.s. (0.941) |
| Dyslipidemia (n, %) | 92 (46.5%) | 29 (47.5%) | n.s. (0.868) |
| Obesity (n, %) | 66 (34.6%) | 20 (34.5%) | n.s. (0.883) |
| Last follow-up | | | |
| Arterial hypertension (n, %) | 149 (74.9%) | 37 (61.7%) | <0.05 |
| Diabetes mellitus (n, %) | 60 (30.2%) | 16 (26.2%) | n.s. (0.691) |
| Dyslipidemia (n, %) | 107 (53.8%) | 27 (44.3%) | n.s. (0.442) |
| Obesity (n, %) | 73 (38.5%) | 21 (35.6) | n.s. (0.195) |

Abbreviations: n.s., not significant.

# Supplemental References

1. Dindo D, Demartines N, Clavien PA. Classification of Surgical Complications: A New Proposal with Evaluation in a Cohort of 6336 Patients and Results of a Survey. *Ann Surg* (2004) 240(2):205-13. Epub 2004/07/27. doi: 10.1097/01.sla.0000133083.54934.ae.
